# The Modern Hospital

**Published:** 1909-09-25

**Authors:** 


### The Hospital

A Journal of

The Medical Sciences and Hospital Administration.

New Series. No. 135, Vol. V. [No. 1207, Vol. XLVI.] SATURDAY, SEPTEMBER 25, 1909.

### The Modern Hospital.

Sir Henry Burdett has been engaged for the past month in making an inspection of the principal hospitals in South, West, and Middle England, and in Wales. He has made a report upon each institution visited, and has been welcomed everywhere with a cordiality and consideration which testify to the vitality of the management and work of the modern hospital all over the country. When Sir Henry Burdett began his hospital work in Birmingham, forty-two years ago, the public was prejudiced against hospitals—a feeling which was justified by the fact that one in three of the patients who underwent major operations died. To-day the mortality in these cases is probably less than three per cent. There was the further objection that trained nurses, as the profession knows them nowadays, were unknown, and the structural, hygienic, and general condition and comfort of the hospitals, according to modern notions, were lamentable in the extreme. Hence the hospitals of those days were places which filled people with dread which no one entered willingly or at all if it could possibly be helped.

These facts make Sir Henry's experience of upwards of forty years as an active hospital worker of very real interest to the profession and the public. Mr. Holmes and Dr. Bristowe inspected, and made a report on, the hospitals at the request of the Privy Council in 1865, three years before Sir Henry took charge of a hospital. During all this period, working in the very centre of hospital life and movement, being consulted by hospital managers in many countries, and having inspected most of the principal hospitals of the world, his present tour of inspection has been welcomed by hospital authorities, and his reports are looked forward to with keen anticipation. They will form a chapter in the history of the development of these institutions which cannot fail to prove of interest and value. We have felt that they may properly be made the occasion of a new departure in medical journalism.

For nearly a quarter of a century The Hospital has led the van in every movement which has tended to promote the improvement and development of hospitals, and has so done much to build up the modern hospital as it is to-day. Each voluntary hospital forms a little republic in itself, where the keenest interest is taken by the many zealous workers who constitute the medical and resident staff, to whose ability and devotion the public owes the security of the modern hospital.

We have decided to commence a new series of The Hospital with our issue of October 2, 1909. We feel it to be due to the workers in our hospitals that they should have a newspaper which will strive to do justice to all who labour for the sick, for in them is vested the splendid privilege of ministering to the alleviation of human suffering with a success which is as glorious as it is remarkable. We shall endeavour to show how the modern hospital is worked, who are the men and women to whom the public owes more than it can ever repay, and how the present success has been secured. We shall further record the developments in treatment, in construction, in administration, and throughout each department, as they occur, so that the workers may be encouraged, the finances of the hospitals improved, and the public enabled to take a more intelligent interest in the administration of hospitals than has yet been possible. We shall make it our business to help every one who is interested in hospital work by enabling them to find in The Hospital the fullest and most up-to-date information, whilst they will be encouraged to send us particulars of all matters in which they are specially engaged or interested, and to ask for information through the Question Box department of The Hospital. Here they will readily ascertain any information of which they may be in search, and so be saved an infinity of time and trouble.

Finally, The Hospital, in its new form, will aim at doing justice to the spirit which animates all the best workers throughout the hospital world, without which no modern hospital can become efficient or continue at its best. The generous and warm welcome which Sir Henry Burdett has received in every hospital he has inspected bears striking testimony to the good-fellowship which prevails throughout the country in hospital matters, and is full of promise for the future. No one can fail to read the reports of the present condition of our hospitals all over the country without gaining a mass of useful information and being cheered on his way to make ever greater and more self-denying efforts to secure the maximum of efficiency in every institution with which he may be connected. We rely upon the co-operation of all hospital workers, for their goodwill and steady support are essential to the success of this new departure. We know by experience that the thousands of men and women who are engaged in hospital work in the present day will be glad to have a newspaper of their own, and to do what they can to make it the helpful friend and trusted counsellor of all who believe that if a thing is worth doing it is worth doing well. To the members of Committees and the Governors of hospitals we believe The Hospital, in its new form, will be found both interesting and helpful, whilst to the medical profession, and, indeed, to all who spend much of their time in hospitals, it will be our endeavour to make it indispensable as a trusted friend. It will be the Editors' first duty to do justice to everybody and to provide that every good work shall find in these columns just and full acknowledgment.

